# Expérience d´intégration de la santé mentale en première ligne de soins en Guinée

**DOI:** 10.11604/pamj.2020.37.107.20351

**Published:** 2020-10-01

**Authors:** Abdoulaye Sow, Bart Criel, Bernard Branger, Michel Roland, Myriam De Spiegelaere

**Affiliations:** 1 Ecole de Santé Publique, Université Libre de Bruxelles, Bruxelles, Belgique,; 2 Université Gamal Abdel Nasser de Conakry, Conakry, Guinée,; 3 Institut de Médecine Tropicale d´Anvers, Kronenburgstraat 43, 2000 Antwerpen, Belgique,; 4 Organisation ESSENTIEL, 11 bis rue Gabriel Luneau 44000 Nantes, France

**Keywords:** Santé mentale, soins de santé primaire, intégration, Guinée, Mental health, mental disorders, health centers, integration, Guinea

## Abstract

**Introduction:**

la faible couverture des services spécialisés et la pénurie en ressources humaines en santé mentale représentent d´immenses défis pour les systèmes de santé en Afrique. L'intégration de la santé mentale dans les soins de santé primaires constitue un complément substantiel et faisable aux services spécialisés. Cette étude rassemble et analyse les données générées dans 5 Centres de santé (CS) ayant intégrés ce paquet de soins en Guinée.

**Méthodes:**

l'étude descriptive porte sur les nouveaux cas de santé mentale entre 2012 et 2017. Les motifs de consultations et les diagnostics posés ont été répertoriés et analysés sur la base des registres de consultation et des dossiers médicaux individuels.

**Résultats:**

au total 4995 patients ont consulté pour un problème de santé mentale, ce qui représente 2,8% des consultations générales (de 0,5 à 7,7% selon les centres). La moyenne d'âge des patients était de 27,9 ans (± 16,1). Les motifs de consultations les plus fréquents étaient les insomnies: 44,4% (n = 2081), les crises convulsives: 39% (n = 1827), les troubles du comportement: 31,9% (n = 1263) et les hallucinations: 26,1% (n = 1224). Les diagnostics les plus fréquemment posés étaient l'épilepsie : 36,8% (n = 1773) et les troubles psychotiques: 33,5% (n = 1613). 88,4% (n = 4418) des patients ont reçu un traitement médicamenteux, le plus souvent combiné avec un soutien psychologique.

**Conclusion:**

l´étude montre que dans le contexte guinéen où l´accès aux soins spécialisés en santé mentale est très limité, les malades mentaux, même atteints de pathologies lourdes, peuvent être suivis dans des Centres de santé par un personnel non spécialiste mais formé en santé mentale.

## Abstract (en)

**Introduction:**

the low coverage of specialized mental health services and the shortage of human resources for mental health are enormous challenges for the health care system in Africa. The integration of mental health support into primary health care is a substantial and feasible complementary intervention to specialized services. We collected and analyzed data from 5 Health Care Centers (HCC) that had integrated this care package in Guinea.

**Methods:**

we conducted a descriptive study of new cases of mental health issues between 2012 and 2017. The reasons for consultations and diagnoses were identified and analyzed on the basis of consultation registries and individual medical records.

**Results:**

a total of 4.995 patients with mental health problems received consultations (2.8% of general consultations; 0.5 -7.7% according to the centers). The average age of patients was 27,9 years (± 16.1). The most common reasons for consultations were: insomnia 44.4% (n = 2081), seizures 39% (n = 1827), behavioral disorders 31.9% (n = 1263) and hallucinations 26.1% (n = 1224). The most common diagnoses were: epilepsy 36.8% (n = 1773) and psychotic disorders 33.5% (n = 1613). Eighty eight point four per cent (n = 4418) of patients received medical treatment, most often combined with psychological support.

**Conclusion:**

this study highlights that in the Guinean context, where access to specialized mental health care is very limited, patients with mental ill, even with severe medical conditions, can be followed up in the health centers by non-specialized but trained mental health professionals.

## Introduction

Les troubles mentaux représentent une charge de morbidité très élevée dans le monde [1]. Ils représentaient 19% de la charge d´incapacité en Afrique (Années de Vie Corrigée du facteur d'Invalidité, AVCI) en 2010 et cette part devrait s´alourdir dans les années qui viennent [2]. Le défi que ces troubles posent aux systèmes de santé africains est donc particulièrement important [3]. En Afrique les enquêtes communautaires montrent que seule une minorité des personnes souffrant de troubles graves de santé mentale bénéficient d´un traitement [4, 5]. Les moyens alloués aux programmes nationaux de santé mentale sont faibles, et généralement orientés vers les hôpitaux psychiatriques, le plus souvent inaccessibles aux plus nécessiteux. Au-delà des structures, la pénurie de moyens touche surtout les ressources humaines spécialisées et les médicaments. On y compte 1,4 professionnel de santé mentale pour 100 000 habitants, contre 9 dans le monde [6].

Face à cette situation, le traitement des troubles mentaux au niveau des soins primaires, est la stratégie la plus souvent avancée et soutenue par L´OMS [3, 4, 6]. De plus en plus d´études évaluent l´efficacité de l´offre de soins en santé mentale dans les services généraux des structures communautaires et services de santé de base comme alternative aux structures hospitalières insuffisantes dans les pays moins avancés [7, 8]. Il en ressort que la prise en charge des troubles mentaux dans les soins de santé primaires avec un dispositif de transfert de compétences des spécialistes vers les agents de santé travaillant à proximité des patients est faisable et acceptable [9], et efficace [10] sous certaines conditions. Cette étude vise à évaluer une expérience d´intégration des soins en santé mentale par la première ligne de soins en Guinée.

La Guinée ne dispose que d´un seul service de psychiatrie situé dans la capitale Conakry, comptant une trentaine de lits d´hospitalisation et 5 psychiatres, sans infirmiers spécialisés ni psychologues, pour près de 11 millions d´habitants. Dans la quasi-totalité du territoire, les soins aux malades mentaux relèvent des seuls guérisseurs traditionnels [11]. C´est dans ce contexte que l´ONG Fraternité Médicale Guinée (FMG), qui gère des Centres de santé (CS) avec des équipes de soins multidisciplinaires dont des médecins de famille, a intégré la prise en charge des malades mentaux dans 3 de ses CS depuis 2000, dans le cadre du projet « Santé Mentale en Milieu Ouvert Africain » (SaMOA) [12]. Elle a ensuite étendu cette intégration dans deux autres CS non FMG. Le paquet de soins offert en santé mentale comprend la consultation, le traitement médicamenteux, l´accompagnement psychosocial et la réhabilitation. Sur base des données de routine collectées par ces 5 CS, cette étude vise à évaluer l´utilisation des soins de santé mentale dans ces centres et à déterminer les caractéristiques des patients et des pathologies rencontrées ainsi que leur prise en charge.

## Méthodes

### Type et cadre de l´étude

Cette étude descriptive et rétrospective, a été réalisée dans 5 CS ayant intégré la santé mentale dans leur paquet de soins: 3 centres de FMG (Hafia Minière, Tata1 et Moriady), et 2 centres non FMG (Timbi Madina et Pita Centre). Deux de ces CS sont situés en zone rurale (Moriady et Timbi Madina), les 3 autres en zone urbaine.

### Population d´étude

Tous les patients ayant consulté dans les 5 CS pour un problème de santé mentale entre le 1^er^ janvier 2012 et le 31 décembre 2017 ont été inclus. Seuls les nouveaux cas (premier contact pour un problème de santé mentale) ont été pris en compte.

### Collecte des données

Le recueil des données a été réalisé à partir des registres de consultation. Les données des dossiers médicaux individuels ont également été utilisées dans les 3 centres FMG qui en disposent. Les données recueillies portaient d´une part sur des informations socio-démographiques et d´autre part sur les caractéristiques médicales : motif de consultation, diagnostic posé, traitements prescrits. L´outil de collecte des données reprenait pour chaque item une liste de choix à cocher (plusieurs réponses possibles), les catégories « autres » ouvrant un champ en texte libre. Les diagnostics ont été recodés en 7 catégories exclusives (schizophrénie et autre troubles délirants, troubles de l´humeur, troubles névrotiques, épilepsies, démence, retard psychomoteur, autres) sur base de la classification internationale des maladies (CIM10), adaptée à la pratique et à la qualification des soignants de première ligne dans le contexte guinéen. Ces données quantitatives ont été complémentées par des informations venant des rapports d´activités de routine des différentes structures de soins concernées par l´étude, ainsi que par le vécu des professionnels travaillant dans ces structures.

### Analyses statistiques

Les données ont été analysées avec le logiciel SPSS 22.0. Les comparaisons de proportions ont été effectuées avec le test du χ^2^ avec un seuil de signification à p < 0.05.

### Considérations éthiques

L´étude a été approuvée par la Commission Nationale d´éthique pour la Recherche en santé (CNERS) de Guinée (N°010/CNRS/17).

## Résultats

Au total 4995 patients se présentant pour la première fois avec un problème de santé mentale ont été enregistrés entre 2012 et 2017 dans les 5 CS. Toutes les données n´étaient pas disponibles pour tous les patients et pour toutes les variables. Les résultats présentés portent sur les données valides et donc les totaux diffèrent selon les analyses.

### Distribution des patients dans les 5 CS

Leur distribution était très inégale entre les centres. Les 3 centres de FMG ont pris en charge 92,6% (n = 4628) des patients. Le Tableau 1 présente le nombre de nouveaux cas de santé mentale par rapport au nombre total de nouveaux cas enregistrés dans chaque centre. Pour l´ensemble des CS, en moyenne 2,8% (n=4995) des consultations étaient en lien avec des problèmes de santé mentale. Cette proportion différait fortement d´un centre à l´autre et d´une année à l´autre.

**Tableau 1:** proportion des patients avec problèmes de santé mentale (SM) parmi l´ensemble des patients des consultations générales des centres de santé

		Hafia manière	Moriady	Tata1	Timbi madina	Pita centre	Total
2012	SM	380	182	241	69	105	977
	Total	11 931	2 853	6 190	4 300	10 789	36 063
	%	3,2%	6,4%	3,9%	1,6%	1,0%	2,7%
2013	SM	260	192	221	16	45	734
	Total	9 789	2 622	6 283	4 260	8 725	31 679
	%	2,7%	7,3%	3,5%	0,4%	0,5%	2,3%
2014	SM	210	184	237	10	20	661
	Total	11 852	3 152	2 921	4 236	9 432	31 593
	%	1,8%	5,8%	8,1%	0,2%	0,2%	2,1%
2015	SM	204	177	226	12	23	642
	Total	9 857	2 623	2 104	4 128	6 333	25 045
	%	2,1%	6,7%	10,7%	0,3%	0,4%	2,6%
2016	SM	456	116	312	30	3	917
	Total	12 128	1 619	2 095	5 328	4 877	26 047
	%	3,8%	7,2%	14,9%	0,6%	0,1%	3,5%
2017	SM	396	190	444	16	18	1 064
	Total	12 616	1 211	2 173	5 532	5 831	27 363
	%	3,1%	15,7%	20,4%	0,3%	0,3%	3,9%
Total	SM	1 906	1 041	1 681	153	214	4 995
	Total	68 173	14 080	21 766	27 784	45 987	177 790
	%	2,8%	7,4%	7,7%	0,6%	0,5%	2,8%

### Caractéristiques sociodémographiques

Les patients étaient âgés de 0 à 98 ans (moyenne 27,9 ans ± 16,1). Le Tableau 2 présente les caractéristiques sociodémographiques de la population d´étude. La situation matrimoniale différait en fonction du genre: par rapport aux hommes, les femmes étaient plus souvent célibataires: 46,7% versus 26,8%, divorcées: 2,4% versus 1,3% ou veuves: 2,3% versus 0,3% (p < 0.001). La répartition des ethnies correspondait à la situation géographique des CS et à la proportion des différents groupes ethniques dans le pays.

**Tableau 2:** caractéristiques sociodémographiques des patients

		N	%
Sexe	Homme	2697	54,1%
	Femme	2286	45,9%
**Total**		**4983**	**100,0%**
Résidence	Urbaine	3786	79,4%
	Rurale	984	20,6%
**Total**		**4770**	**100%**
Ethnie	Peulh	2595	72,0%
	Soussou	484	13,4%
	Malinké	426	11,8%
	Forestier	64	1,8%
	Autres	37	1,0%
**Total**		**3606**	**100%**
Situation familiale	Célibataire	1408	35,80%
	Marié	2413	61,30%
	Divorcé	70	1,80%
	Veuf	46	1,20%
**Total**		**3937**	**100%**

### Motifs de consultation

**Figure 1:**
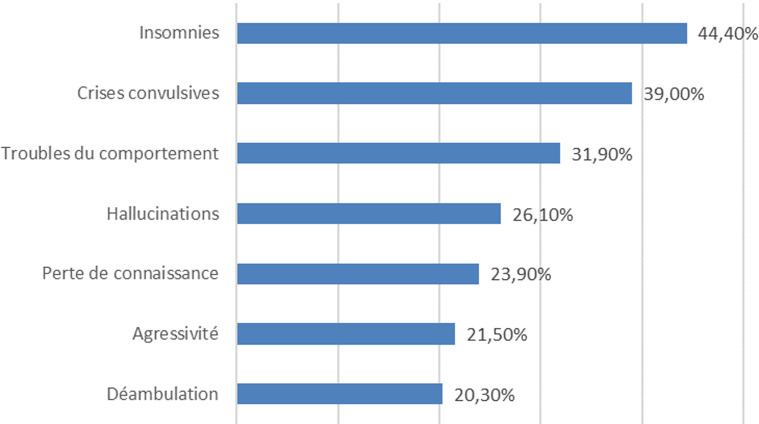
principaux motifs de consultation

La Figure 1 montre les principaux motifs de consultations. Très peu de patients, 2,8% (n = 126), consultaient pour consommation de substances psychoactives. Les symptômes étaient le plus souvent multiples, plus de la moitié des patients (53%, n=2647) présentaient 4 symptômes ou plus.

### Diagnostic

Le Tableau 3 présente la fréquence des principaux diagnostics. L´épilepsie est le diagnostic le plus fréquent, suivi par les psychoses qui représentent un tiers des cas. La catégorie « autres » comprenait des retards psychomoteurs, les démences et d´autres problèmes psychosociaux. La distribution des diagnostics posés différait significativement entre les CS (p < 0.001). En particulier, on observait une fréquence plus élevée de diagnostic d´épilepsies dans le CS Moriady et de troubles psychotiques dans les CS non-FMG (Timbi Madina et Pita).

**Tableau 3:** principaux diagnostics posés par centres de santé

	Hafia-Minière	Moriady	Tata1	Timbi-Madina	Pita Centre	Total
Epilepsies	604	531	522	49	67	1773
	33,2%	52,8%	31,9%	32,9%	31,8%	36,8%
Schizophrénie et autres troubles délirants	602	221	606	71	113	1613
	33,1%	22,0%	37,1%	47,7%	53,6%	33,5%
Troubles de l´humeur	308	155	241	7	8	719
	16,9%	15,4%	14,7%	4,7%	3,8%	14,9%
Troubles névrotiques, liés au stress et somatoformes	134	29	179	5	2	349
	7,4%	2,9%	11,0%	3,4%	0,9%	7,2%
Autres	171	70	86	17	21	365
	9,4%	7,0%	5,3%	11,4%	10,0%	7,6%
Total	1819	1006	1634	149	211	4819
	100,0%	100,0%	100,0%	100,0%	100,0%	100,0%

### Traitement

La plupart des patients, 88,4% (n = 4260), avaient reçu un traitement médicamenteux. Cette proportion différait significativement selon le diagnostic posé: 97,6% (n = 702) des troubles de l´humeur, 96,7% (n = 1560) des psychoses et 95,9% (n = 1701) des épilepsies avaient été traités par médicaments pour 25,1% (n=88) des troubles névrotiques et 90,1% (n=329) des diagnostics « autres » (p < 0.001). L´Halopéridol est le médicament le plus souvent administré. Les médicaments utilisés sont présentés dans le Tableau 4. Les médicaments étaient le souvent combinés avec d´autres approches thérapeutiques. Ainsi, parmi les patients ayant reçu au moins 1 médicament, 98,6% (n = 3483) avaient bénéficié d´un soutien psychologique, 20,8% (n = 682) d´une assistance sociale et 7,6% (n = 337) d´une réinsertion.

**Tableau 4:** principaux médicaments administrés

Halopéridol	48,5%
**Carbamazépine**	36,6%
**Biperiden**	33,3%
**Diazépam**	32,4%
**Amitripline**	15,3%
**Chlorpromazine**	14,2%
**Autres**	11,8%
**Placebo**	7,8%
**Magnésium/vitamine B6**	7,2%
**Lévomépromazine**	3,4%
**Clonazepam**	1,9%

## Discussion

Cette étude montre le potentiel important des CS pour améliorer la couverture des besoins en santé mentale en Afrique. L´évaluation du système national de santé mentale réalisée en Guinée montrait qu´en 2014 le service de psychiatrie de l´hôpital national Donka, seul service spécialisé public du pays, avait admis 1264 patients en consultation ambulatoire [11], alors que, pour la même année, les 5 CS objets de la présente étude avaient pris en charge 661 nouveaux cas en santé mentale, soit 52% du nombre de cas suivis par Donka pour une zone couvrant environ 40 000 habitants. Avant l´intégration de la santé mentale dans les 5 CS, toutes les personnes ayant des troubles mentaux étaient référées au service de psychiatrie de l´hôpital national. Nos résultats montrent que la prise en charge de la santé mentale dans les CS, est faisable et permet d´améliorer la couverture des services de soins en santé mentale, ce qui rejoint les conclusions d´autres études [10]. Dans notre étude, la part des consultations pour un problème de santé mentale représente 2,8% de l´ensemble des consultations, alors qu´au Québec, un tout autre contexte avec une couverture en soins de santé mentale bien plus adéquate, 25% des visites chez les médecins de famille sont liées à des troubles mentaux et 24% dans une autre étude sur 15 sites [13-16].

Cette part semble donc faible et varie fortement d´un centre à un autre et d´une année à l´autre (0,2 à 20,4%). Elle s´explique par différents facteurs. Tout d´abord, seuls les premiers contacts ont été comptabilisés. Les troubles mentaux sont chroniques et impliquent en général un nombre élevé de contacts, ce qui peut expliquer la part importante de l´activité de médecine générale si l´on considère l´ensemble des consultations réalisées par les médecins de famille. D´autre part, dans le contexte guinéen, les patients ne consultent pas pour certains troubles mentaux qu´ils jugent relevé des guérisseurs traditionnels. Enfin, nous avons pris en compte l´ensemble des consultations primaires curatives alors que, dans certains CS, seules certaines consultations assurées par des professionnels formés offraient ces soins. Les différences entre CS sont partiellement dues à la chronologie de l´intégration de la santé mentale entre 2000 (Hafia Minière) et 2012 (Tata1). En général, les premières années après l´intégration, le nombre de consultations augmente progressivement avant de se stabiliser, voire régresser. La part croissante des pathologies mentales dans le CS Tata1 est liée à sa position géographique. Ce centre couvre en effet toute une région sanitaire et étant le seul à offrir les soins de santé mentale dans cette région, il est perçu comme un « Centre de santé mentale ».

Pour les centres non-FMG, le nombre de cas est plus important la première année du fait de la publicité et de l´effet de nouveauté et diminue ensuite. Les variations dans le temps peuvent être liées aussi aux mouvements du personnel, fréquents dans le secteur public, comme au CS de Pita. Plusieurs autres facteurs, documentés dans les rapports d´activités des centres de santé, interviennent tels que les ruptures de médicaments, l´arrivée de nouveaux guérisseurs traditionnels ou l´épidémie d´Ebola. Au cours du temps, par la nécessité d´un suivi à long terme, le nombre de patients suivis augmente ainsi que la charge de travail. Ceci implique que la part de nouveaux cas en santé mentale dans les consultations ne reflète pas forcément la part de la charge de travail qui y est consacrée. Selon les rapports d´activités des centres de santé, au début du processus d´intégration, les malades viennent de la zone de couverture des CS, mais au fil des ans, ils viennent de zones de plus en plus éloignées grâce au bouche à oreille et aux succès thérapeutiques constatés.

Concernant les caractéristiques des patients, toutes les tranches d´âge sont représentées. Même si le nombre de divorcés est peu important, les femmes sont les plus concernées. Dans le contexte guinéen en effet, quand la maladie mentale survient dans un foyer, le couple finit souvent par se disloquer et généralement, si les femmes ne sont pas « rendues » à leurs parents, elles sont abandonnées par leur conjoint. L´insomnie ressort comme le principal motif de consultation pour les patients avec un problème de santé mentale. Ce symptôme étant également fréquemment retrouvé dans de nombreuses pathologies physiques, l´intégration de la santé mentale dans l´offre des soins de première ligne constitue une opportunité pour sensibiliser les soignants des CS à la prise en compte des aspects psychosociaux dans leur consultation de médecine générale. Nos résultats montrent que toutes les pathologies psychiatriques rencontrées dans les services spécialisés sont susceptibles d´être identifiées dans les CS, que ce soit les pathologies psychiatriques lourdes comme les psychoses et la dépression ou certaines maladies neurologiques comme les épilepsies, les démences et les retards psychomoteurs.

On note que la distribution des diagnostics varie entre CS. Au CS de Moriady, plus de la moitié des cas sont des épilepsies. Ceci s´explique par les activités communautaires plus importantes qui ont été réalisées autour du CS, avec l´implication de la radio rurale qui couvre plusieurs sous-préfectures et a longuement parlé des épilepsies dans les langues nationales. Tous les CS publics de Kindia y réfèrent également les épileptiques sur recommandation du médecin chef de district (Communication personnelle du médecin chef de district de Kindia). Dans les CS non FMG, les pathologies psychotiques sont les plus représentées. L´une des hypothèses est la présence plus importante de ce type de patients dans les petites agglomérations (petites villes ou villages) où sont situés ces CS. Lorsque les familles sont épuisées dans le parcours de soins avec les malades mentaux en ville, elles préfèrent les envoyer au village où ils seraient plus faciles à canaliser avec des méthodes traditionnelles de contention (Communication personnelle des responsables des centres de santé).

Si les médicaments restent les moyens thérapeutiques les plus utilisés, l´accompagnement psychosocial et la réinsertion, pour lesquels les soignants ont été formés dans le processus d´intégration, apportent une valeur ajoutée dans l´offre de soins en première ligne. Des placebos sont aussi utilisés pour réduire la consommation des médicaments souvent exigés par les familles des patients. Les difficultés rencontrées lors du recueil des informations quantitatives limitent l´interprétation de certains résultats. Les sources de données n´étaient pas homogènes et dépendaient des outils de routine utilisés par les différents centres. Les trois CS FMG utilisaient, en plus des registres standards, des dossiers médicaux individuels ainsi que des cahiers de visite à domicile et de réinsertion des patients. Les deux autres CS n´utilisaient que les registres de consultation. La faible qualité du remplissage et l´absence de dossiers médicaux expliquent l´importance des données manquantes et/ou aberrantes pour certaines variables dans ces CS.

Pour les centres disposant de dossiers médicaux, le recueil de données ne s´est pas toujours limité aux informations du premier contact avec le patient, des informations provenant des documents du suivi du patient ont été recueillies expliquant dans certains cas des diagnostics et traitements multiples à première vue incohérents. Toutefois, cette proportion de patients reste marginale. Du fait de la faible qualité des données des registres, nous n´avons pu obtenir certaines informations, comme la proportion de malades améliorés, des perdus de vue et de rechutes.

Une étude plus approfondie devrait se pencher sur ces dimensions de la qualité des soins sur base d´un suivi longitudinal, et donc la tenue de dossiers individuels [10]. Cette étude montre qu'il est possible d´analyser, sur base de données de routine, l´utilisation des soins de santé mentale en première ligne des soins dans un pays d´Afrique lorsque ceux-ci y sont offerts. Pour réellement évaluer l´amélioration de la couverture des besoins il est toutefois indispensable de disposer de données plus spécifiques sur les besoins dans la population générale et leur évolution dans le temps [15].

## Conclusion

Notre expérience montre que l´intégration de la santé mentale en première ligne de soins est possible et utile: les patients utilisent les CS qui peuvent être des lieux « adéquats » pour offrir des soins aux patients ayant des pathologies neuropsychiatriques lourdes. Les médicaments essentiels génériques, l´accompagnement psychologique et une réinsertion apportent des réponses efficaces à un nombre important de problèmes de santé mentale. Des enquêtes communautaires devraient être menées pour estimer les besoins en santé mentale dans les zones desservies par ces centres de manière à évaluer et progressivement améliorer la couverture des soins.

### Etat des connaissances sur le sujet

- La morbidité liée aux troubles mentaux est élevée dans tous les pays;
- La disponibilité des soins de santé mentale en Afrique est marginale;
- Plusieurs études confirment l´efficacité des services de santé de base comme modalité de prise en charge des malades mentaux.

### Contribution de notre étude à la connaissance

- Quand les soins en santé mentale sont intégrés dans les centres de santé en Guinée, ils sont utilisés par la population;
- Du fait du contexte local, des pathologies neurologiques graves comme les épilepsies et les démences peuvent se retrouver dans la sphère des maladies mentales;
- En cas de pénurie en ressources humaines et de structures spécialisées en santé mentale, les CS polyvalents peuvent être une alternative valable pour améliorer l´accessibilité aux soins pour les personnes ayant des troubles mentaux.

## Remerciements

Nous remercions le Pr Keïta Mamady Mory pour le protocole, Isabelle Godin pour les analyses statistiques; Hamidou Diallo et Youssouf Baldé pour la récolte des données, les soignants des 5 CS pour la facilité de recueil et Monique Van Dormael de l´Institut de Médecine Tropicale d´Anvers et Mamadou Pathé Diallo de l´Université de Conakry pour la relecture du manuscrit.
